# Effects of cerebral small vessel disease on the outcomes in cryptogenic stroke with active cancer

**DOI:** 10.1038/s41598-021-97154-1

**Published:** 2021-09-01

**Authors:** Ki-Woong Nam, Hyung-Min Kwon, Yong-Seok Lee, Jeong-Min Kim, Sang-Bae Ko

**Affiliations:** 1 Department of Neurology, Seoul Metropolitan Government-Seoul National University Boramae Medical Center, Seoul, South Korea; 2 Department of Neurology, Seoul National University College of Medicine, Seoul Metropolitan Government-Seoul National University Boramae Medical Center, 20 Boramae-ro 5-gil, Dongjak-Gu, Seoul, 07061 South Korea; 3 Department of Neurology, Seoul National University College of Medicine, Seoul National University Hospital, 101 Daehakno, Jongno-Gu, Seoul, 03080 South Korea

**Keywords:** Cancer, Neurology, Neurological disorders

## Abstract

Cerebral small vessel diseases (cSVDs) affect the prognosis of various types of ischemic stroke. Therefore, we evaluated the association between cSVD and the prognosis of cryptogenic stroke patients with active cancer. We enrolled patients diagnosed with cryptogenic stroke and active cancer from 2010 to 2016. Early neurological deterioration (END) was defined as a ≥ 2-point increase in the total NIHSS score or a ≥ 1-point increase in the motor NIHSS score within the first 72 h. We defined an unfavorable outcome as the modified Rankin Scale (mRS) score ≥ 3 points. We analyzed cSVD separately for each subtype including white matter hyperintensity (WMH), silent brain infarct (SBI), and cerebral microbleed (CMB). A total of 179 cryptogenic stroke patients with active cancer were evaluated. In the multivariable analysis, SBI was significantly associated with END (adjusted odds ratio = 3.97, 95% confidence interval: 1.53–10.33). This close relationship between SBI and END increased proportionally with an increase in SBI burden. However, WMH and CMB showed no significant association with END. None of the cSVD subtypes showed a statistically significant relationship with the 3-month unfavorable outcome. SBI was the only parameter closely associated with END in cryptogenic stroke patients with active cancer.

## Introduction

Ischemic stroke and cancer are common diseases and leading causes of disability and death worldwide^1,2^. Recently, active cancer has been recognized as a major risk factor for ischemic stroke^1–4^. Cancer is commonly found in up to 15% of patients with ischemic stroke^1,3,5^. In addition, stroke in patients with cancer is more severe, recurs frequently, and has a poor prognosis^3,6–9^.

Over the decades, remarkable advances have been made in the diagnosis and treatment of cancer, increasing the life expectancy of patients with cancer^1,3,10^. Therefore, the long-term quality of life of cancer patients is now becoming an important issue^10^. Moreover, oncologists tend to hesitate during cancer treatment because patients may not be able to tolerate the side effects of chemotherapy because of post-stroke disability^3^. Thus, it is important to minimize disability by preventing early recurrence in ischemic stroke patients with active cancer. The mechanism of ischemic stroke is more complex in patients with cancer than in those without cancer^1,5,11^. Therefore, it is necessary to study their stroke mechanism and risk factors. Previous studies have identified the cryptogenic mechanism, initial stroke severity, neutrophil-to-lymphocyte ratio, D-dimer level, and multiple territory to be risk factors^5–7,9,11–14^.

Cerebral small vessel disease (cSVD) is a sub-clinical condition encompassing diverse pathologies such as white matter hyperintensity (WMH), silent brain infarct (SBI), and cerebral microbleeds (CMBs)^15^. Despite the different pathological morphologies, cSVD subtypes often coexist^15^. Additionally, an increase in the total cSVD burden leads to a vulnerable brain environment^15–17^. In previous studies, a high cSVD burden increased the risks of vascular dementia, ischemic stroke, and mortality^15,17^. Particularly, cSVD is closely related to the prognosis of ischemic stroke through various mechanisms, such as large artery disease or cardioembolism in addition to lacunar stroke, which has the same mechanism^18–21^. Therefore, cSVD is believed to have a significant effect on the prognosis of stroke patients with active cancer. However, no research has been conducted on this topic.

The aim of the present study was to investigate the association between cSVD and the prognosis of cryptogenic stroke patients with active cancer. Further, we aimed to elucidate the relationship between cSVD subtypes and the prognosis in these patients. Finally, based on these comparisons, we aimed to determine whether the influence of cSVD subtypes on the prognosis resulted from the general pathological mechanism of cSVD or from a mechanism related to a specific subtype.

## Results

A total of 179 cryptogenic stroke patients with active caner were evaluated (mean age: 67 ± 10 years, male sex: 60.3%, initial National Institutes of Health Stroke Scale [NIHSS] score: 7 ± 6). Early neurological deterioration (END) events were identified in 31 (17.3%) patients, and the frequency of unfavorable outcomes was 88 (49.2%). We found SBI in 65 (36.3%) patients; single lesions in 26 (14.5%) patients; and multiple lesions in 39 (21.8%) patients. Other detailed baseline characteristics are presented in Supplementary Table 1.

**Figure 1:**
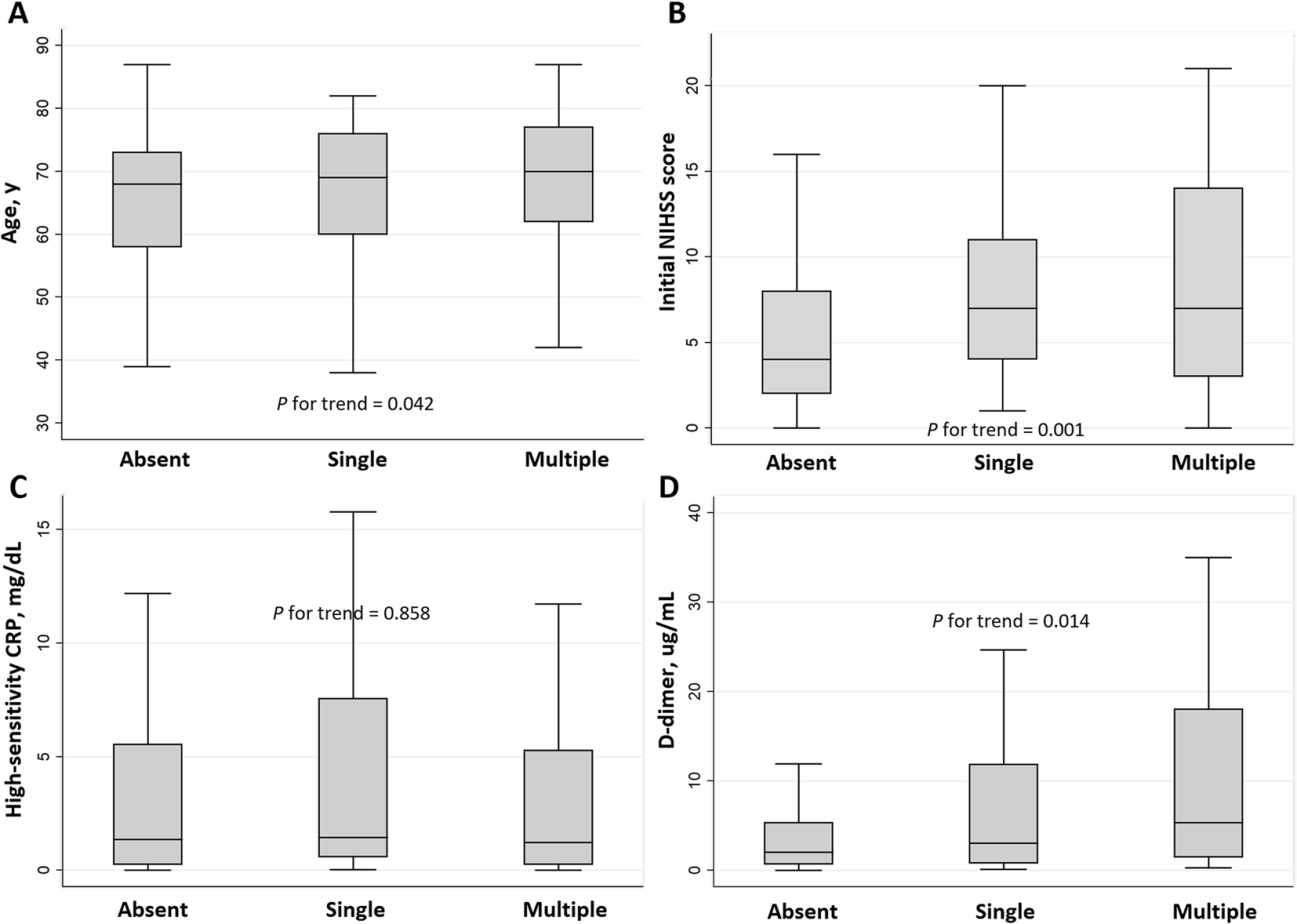
Comparisons of characteristics according to the silent brain infarct burden. Patients with multiple silent brain infarct (SBI) lesions were older (*P* for trend = 0.042), showed higher initial NIHSS score (*P* for trend = 0.001) and D-dimer levels (*P* for trend = 0.014) than those with an no lesion or a single lesion. The SBI burden showed no statistically significant correlation with high-sensitivity C-reactive protein levels.

In the univariate analysis, END was significantly associated with the initial NIHSS score, thrombolytic therapy, D-dimer level, multiple territory lesions, and SBI (Table 1). In the multivariable logistic regression analysis, SBI remained significant after adjusting for confounders [adjusted odds ratio (aOR) = 3.97, 95% confidence interval (CI) 1.53–10.33]. The initial NIHSS score (aOR = 1.09, 95% CI 1.01–1.17) and D-dimer level (aOR = 1.45, 95% CI 1.02–2.06) correlated positively with END, independent of SBI (Table 2). The close relationship between SBI and END increased proportionally with an increase in the SBI burden (single lesion, aOR = 3.05, 95% CI 0.91–10.26; multiple lesions, aOR = 4.89, 95% CI 1.60–14.94). In the comparison of characteristics according to the SBI burden, a higher SBI burden was positively associated with older age, higher initial NIHSS score, and higher D-dimer levels (Fig. 1 and Supplementary Table 2).

**Table 1:** Baseline characteristics of patients with and without early neurological deterioration.

	No END (n = 148)	END (n = 31)	*P* value
Age, y [IQR]	69 [62–74]	64 [58–72]	0.139
Sex, male, n (%)	93 (62.8)	15 (48.4)	0.135
Hypertension, n (%)	66 (44.6)	11 (35.5)	0.352
Diabetes, n (%)	45 (30.4)	10 (32.3)	0.839
Dyslipidemia, n (%)	60 (40.5)	16 (51.6)	0.257
Current smoking, n (%)	50 (33.8)	11 (35.5)	0.856
Cancer type, n (%)			0.605
Lung	46 (31.1)	6 (19.4)	
Gastric/esophageal	14 (9.5)	3 (9.7)	
Colorectal	8 (5.4)	2 (6.5)	
Hepatobiliary	43 (29.1)	12 (38.7)	
Genitourinary	28 (18.9)	8 (25.8)	
Breast	4 (2.7)	0 (0)	
Others	5 (3.4)	0 (0)	
Systemic metastasis, n (%)	85 (57.4)	19 (61.3)	0.692
Adenocarcinoma, n (%)	72 (55.8)	21 (75.0)	0.061
Initial NIHSS score [IQR]	4 [2–7]	10 [6–17]	< 0.001
Thrombolytic therapy, n (%)	7 (4.7)	6 (19.4)	0.004
HbA1c, % [IQR]	6.0 [5.6–6.5]	6.2 [5.8–6.8]	0.095
Fasting glucose, mg/dL [IQR]	101 [88–110]	108 [88–135]	0.101
Total cholesterol, mg/dL [IQR]	159 [127–193]	176 [148–213]	0.078
White blood cell, × 10^3^/µL [IQR]	7.37 [5.68–10.33]	7.96 [6.03–10.36]	0.299
High sensitivity CRP, mg/dL [IQR]	0.94 [0.18–5.54]	2.29 [0.57–8.83]	0.082
D-dimer, µg/mL [IQR]	1.89 [0.68–5.34]	11.44 [2.66–21.07]	< 0.001
MRI lesion pattern, n (%)			0.024
Single territory	76 (51.4)	9 (29.0)	
Multiple territory	72 (48.6)	22 (71.0)	
Periventricular WMH (Fazekas scale), [IQR]	1 [0–2]	1 [0–2]	0.562
Subcortical WMH (Fazekas scale), [IQR]	0 [0–1]	0 [0–0]	0.314
Severe WMH, n (%)	31 (20.9)	5 (16.1)	0.543
Silent brain infarct, n (%)	45 (30.4)	20 (64.5)	< 0.001
Cerebral microbleeds, n (%)	33 (22.3)	8 (25.8)	0.672

END = early neurological deterioration, NIHSS = National Institutes of Health Stroke Scale, CRP = c-reactive protein, MRI = magnetic resonance imaging, WMH = white mater hyperintensity.

**Table 2:** Multivariable logistic regression for possible predictors of early neurological deterioration.

	Crude OR (95% CI)	*P*-value	Adjusted OR (95% CI)	*P*-value
**Model 1**				
Age	0.97 [0.94–1.01]	0.150	0.97 [0.93–1.01]	0.133
Sex	0.55 [0.25–1.21]	0.138	0.53 [0.20–1.36]	0.184
Initial NIHSS score	1.14 [1.07–1.21]	< 0.001	1.09 [1.01–1.17]	0.027
D-dimer*	1.80 [1.35–2.42]	< 0.001	1.45 [1.02–2.06]	0.040
Multiple territory lesion	2.58 [1.11–5.98]	0.027	1.03 [0.36–2.92]	0.963
Silent brain infarct	4.16 [1.84–9.40]	0.001	3.97 [1.53–10.33]	0.005
**Model 2**				
Age	0.97 [0.94–1.01]	0.150	0.97 [0.93–1.01]	0.124
Sex	0.55 [0.25–1.21]	0.138	0.51 [0.19–1.34]	0.172
Initial NIHSS score	1.14 [1.07–1.21]	< 0.001	1.08 [1.01–1.17]	0.028
D-dimer*	1.80 [1.35–2.42]	< 0.001	1.45 [1.02–2.06]	0.040
Multiple territory lesion	2.58 [1.11–5.98]	0.027	1.02 [0.36–2.90]	0.976
Silent brain infarct		0.002		0.015
No	Ref	Ref	Ref	Ref
Single	3.45 [1.19–10.02]	0.023	3.05 [0.91–10.26]	0.071
Multiple	4.68 [1.88–11.64]	0.001	4.89 [1.60–14.94]	0.005

NIHSS = National Institutes of Health Stroke Scale.

*These variables were transformed using a log scale.

According to the results of the univariate analysis, unfavorable outcomes were closely associated with male sex, type of cancer, systemic metastasis, adenocarcinoma, the initial NIHSS score, thrombolytic therapy, hemoglobin A1c level, fasting glucose level, high-sensitivity C-reactive protein (hs-CRP) level, D-dimer level, multiple territory lesions, subcortical WMH, SBI, and CMBs (Table 3). However, SBI, WMH, and CMB were not significant in the multivariable logistic regression analyses (Table 4). Unfavorable outcomes were closely related to systemic metastasis (aOR = 2.82, 95% CI 1.03–7.72), the initial NIHSS score (aOR = 1.27, 95% CI 1.10–1.47), and multiple territory lesions (aOR = 3.01, 95% CI 1.10–8.19).

**Table 3:** Baseline characteristics between favorable and unfavorable outcome groups.

	Favorable outcome (n = 80)	Unfavorable outcome (n = 88)	*P* value
Age, y [IQR]	68 [62–74]	69 [61–74]	0.990
Sex, male, n (%)	55 (68.8)	47 (53.4)	0.042
Hypertension, n (%)	38 (47.5)	35 (39.8)	0.313
Diabetes, n (%)	21 (26.2)	29 (33.0)	0.342
Dyslipidemia, n (%)	40 (50.0)	31 (35.2)	0.053
Current smoking, n (%)	29 (36.2)	31 (35.2)	0.890
Cancer type, n (%)			< 0.001
Lung	27 (33.8)	20 (22.7)	
Gastric/esophageal	5 (6.2)	12 (13.6)	
Colorectal	5 (6.2)	5 (5.7)	
Hepatobiliary	13 (16.2)	39 (44.3)	
Genitourinary	22 (27.5)	11 (12.5)	
Breast	3 (3.8)	1 (1.1)	
Others	5 (6.2)	0 (0)	
Systemic metastasis, n (%)	32 (40.0)	64 (72.7)	< 0.001
Adenocarcinoma, n (%)	33 (45.2)	53 (71.6)	0.001
Initial NIHSS score [IQR]	3 [1–5]	9 [4–14]	< 0.001
Thrombolytic therapy, n (%)	1 (1.2)	11 (12.5)	0.005
HbA1c, % [IQR]	5.8 [5.5–6.3]	6.1 [5.8–6.5]	0.004
Fasting glucose, mg/dL [IQR]	99 [84–108]	105 [91–117]	0.022
Total cholesterol, mg/dL [IQR]	159 [138–205]	171 [131–191]	0.619
White blood cell, × 10^3^/µL [IQR]	6.85 [5.67–9.65]	8.01 [6.02–10.40]	0.067
High sensitivity CRP, mg/dL [IQR]	0.50 [0.08–1.46]	2.79 [0.90–9.04]	< 0.001
D-dimer, µg/mL [IQR]	0.97 [0.35–2.70]	4.84 [1.57–18.02]	< 0.001
MRI lesion pattern, (%)			< 0.001
Single territory	55 (68.8)	26 (29.5)	
Multiple territory	25 (31.2)	62 (70.5)	
Periventricular WMH (Fazekas scale), [IQR]	1 [1, 2]	1 [0–2]	0.103
Subcortical WMH (Fazekas scale), [IQR]	0 [0–1]	0 [0–0]	0.018
Severe WMH, n (%)	19 (23.8)	16 (18.2)	0.375
Silent brain infarct, n (%)	23 (28.8)	40 (45.5)	0.026
Cerebral microbleeds, n (%)	26 (32.5)	15 (17.0)	0.020

NIHSS = National Institutes of Health Stroke Scale, CRP = c-reactive protein, MRI = magnetic resonance imaging, WMH = white matter hyperintensity.

**Table 4:** Multivariable logistic regression analysis to evaluate the effects of cerebral small vessel disease on unfavorable outcome (mRS 3–6).

	Crude OR (95% CI)	*P*-value	Adjusted OR (95% CI)	*P*-value
**Model 1**				
Age	1.00 [0.97–1.03]	0.735	1.01 [0.96–1.07]	0.601
Sex	0.52 [0.28–0.98]	0.043	0.70 [0.26–1.90]	0.481
Systemic metastasis	4.00 [2.09–7.65]	< 0.001	2.82 [1.03–7.72]	0.044
Adenocarcinoma	3.06 [1.54–6.06]	0.001	1.67 [0.63–4.44]	0.302
Initial NIHSS score	1.32 [1.20–1.45]	< 0.001	1.27 [1.10–1.47]	0.001
Fasting glucose*	3.92 [1.09–14.16]	0.037	1.78 [0.35–8.97]	0.484
High-sensitivity CRP*	1.70 [1.39–2.07]	< 0.001	1.34 [0.99–1.80]	0.058
D-dimer*	2.38 [1.76–3.20]	< 0.001	1.24 [0.81–1.89]	0.318
Multiple territory lesion	5.25 [2.72–10.13]	< 0.001	3.01 [1.10–8.19]	0.031
Silent brain infarct	2.07 [1.09–3.92]	0.027	1.57 [0.52–4.68]	0.422
**Model 2**^†^				
Severe WMH	0.71 [0.34–1.51]	0.376	1.69 [0.46–6.22]	0.431
**Model 3**^†^				
Cerebral microbleeds	0.43 [0.21–0.88]	0.022	0.33 [0.10–1.08]	0.067

mRS = modified Rankin Scale, NIHSS = National Institutes of Health Stroke Scale, CRP = c-reactive protein, WMH = white matter hyperintensity.

*These variables were transformed using a log scale.

^†^Adjusted for age and *P* < 0.05 in univariate analysis (sex, systemic metastasis, adenocarcinoma, initial NIHSS score, fasting glucose, high-sensitivity CRP, D-dimer, and multiple territory lesion).

In the analysis of subsequent outcomes, the END group exhibited a higher discharge NIHSS score (15 [6–26] vs. 2 [1–6], *P* < 0.001), discharge (5 [5, 6] versus 2 [1–5], *P* < 0.001) and 3-month (5 [3–5] versus 2 [1–3], *P* < 0.001) modified Rankin Scale (mRS) score, and more frequent unfavorable outcomes (90.3% versus 43.8%, *P* < 0.001) than the no-END group (Table 5).

**Table 5:** Outcomes between patients with and without early neurological deterioration.

	No END (n = 148)	END (n = 31)	*P* value
Discharge NIHSS score [IQR]	2 [1–6]	15 [6–26]	< 0.001
Discharge mRS score [IQR]	2 [1–5]	5 [5, 6]	< 0.001
3-months mRS score [IQR]	2 [1–3]	5 [3–5]	< 0.001
3-months outcomes, n (%)			< 0.001
Favorable (mRS 0–2)	77 (56.2)	3 (9.7)	
Unfavorable (mRS 3–6)	60 (43.8)	28 (90.3)	

END = early neurological deterioration, NIHSS = National Institutes of Health Stroke Scale, mRS = modified Rankin Scale.

## Discussion

In this study, we demonstrated that SBI was associated with END in cryptogenic stroke patients with active cancer. Since no close association of WMH or CMBs with END was observed, it is believed that an SBI-specific mechanism was involved rather than any pathologic mechanism common to different cSVD subtypes. Moreover, SBI seemed to have sufficient influence to induce END during the acute period, but it was not sufficient to determine the 3-month functional status.

As mentioned earlier, SBI was closely related to END, and the frequency of END increased proportionally with an increase in the number of SBI lesions. These results could be explained by several plausible hypotheses. First, SBI may be a surrogate marker for a high-risk group prone to additional thromboembolism. Cancer-related stroke can result from various mechanisms^1,11,22^. Among them, intravascular thrombosis due to hypercoagulability and proximal embolism due to nonbacterial thrombotic endocarditis are considered to be the main mechanisms^1,3,22^. In our data, 94 (52.5%) patients showed their stroke lesions in multiple vascular territories, and the average D-dimer level was 7.94 ug/mL. Consistent with previous studies, this indicated that stroke due to thromboembolism occurs frequently in patients with active cancer. Moreover, D-dimer levels increased proportionally with an increase in the number of SBI lesions (Fig. 1). This means that SBI was also caused by thromboembolism and shares its pathology with index stroke. In other words, SBI could be interpreted as evidence of a previous embolic event, and lesions occurring at different times are well-known high-risk factors for recurrence in patients with embolic stroke^17^. Second, the index stroke severity may connect the two. The initial NIHSS score is the strongest known risk factor for END. In our study, the initial NIHSS score was closely related to SBI and increased proportionally with an increase in the number of lesions. Thus, SBI may simply be a surrogate indicator of severe stroke causing END. Last, the general effects of cSVD on the prognosis of stroke may be considered. cSVD is related to chronic hypoxia, endothelial dysfunction, and impaired glymphatic pathway, thus creating a vulnerable brain environment^15,16^, in which permanent stroke lesions could easily develop even after subtle ischemic insults. Consequently, the final size of the lesion tends to increase. In addition, a high cSVD burden can inhibit functional recovery by disrupting neural connectivity and cause delayed neuronal death, resulting in END^23^. However, other cSVD pathologies, such as WMH or CMB, were not related to END. Moreover, SBI showed no significant association with subclinical inflammation commonly observed in vulnerable conditions resulting from cSVD. Therefore, the influence of these mechanisms is unlikely to be significant.

While SBI was closely related to END, it did not have a significant impact on the 3-month functional status. SBI was associated with unfavorable outcomes in the univariate analysis but showed no significance in the multivariable analysis. Moreover, systemic metastasis, the initial NIHSS score, and multiple territory lesions were significantly associated with unfavorable outcomes. Cancer-related stroke usually occurs in advanced cancer and has a short median life expectancy^9–11,14^. Therefore, the underlying cancer status naturally plays an important role in determining the subsequent prognosis^9^. In addition, these patients tend to have severe strokes^1,6^. Even if the stroke does not recur, it is believed that the index stroke itself can exert a long-lasting influence. As mentioned earlier, multiple territory lesions are associated with a high severity of cancer-related thromboembolic conditions and can therefore be interpreted as an indicator of the risk of stroke recurrence. These results suggest that SBI affects the prognosis only in the acute period and has no effect on the functional status subsequently. Therefore, it may be interpreted that SBI does not mean much. However, the END group clearly showed poorer discharge and 3-month prognosis than the no-END group (Table 5). In addition, END clearly affected unfavorable outcomes (aOR = 5.28, 95% CI 1.02–27.24), even after adjusting for several confounders (Table 4). Therefore, if we classify the high-risk END group using SBI during the acute period and prevent the occurrence of END through close monitoring and intensive treatment, it will significantly influence the prognosis afterwards.

This study had several limitations. First, this was a retrospective cross-sectional study. The association between SBI and END observed in our study does not guarantee causality. Further prospective studies are required to confirm the causality. Second, information related to the mechanism of END could not be obtained. The analysis of the results of follow-up magnetic resonance imaging (MRI), microembolic signals on transcranial Doppler sonography, and transesophageal echocardiography would have helped elucidate the mechanisms underlying the association between SBI and END. Third, we used a relatively sensitive definition of END^24^. However, as observed in previous studies^6^, our END exhibited a sufficient influence on the subsequent prognosis. Last, we analyzed only the patients who visited within 72 h of symptom onset, considering the definition of END. END occurs frequently early after the index stroke. Therefore, considering cases of END occurring before the visit, the incidence of END might have been underestimated.

SBI was associated with END in cryptogenic stroke patients with active cancer. Among several cSVD subtypes, only SBI showed an association with END. Therefore, special mechanisms related to SBI (e.g., thromboembolism) may have been involved. Classifying the high-risk group using SBI and providing appropriate treatment during the acute period may prevent END and have a good effect on the subsequent prognosis. However, further prospective studies are required to validate our results.

## Methods

### Study population

As part of a consecutive registry of two large medical centers in Korea (Seoul Metropolitan Government-Seoul National University Boramae Medical Center [SMG-SNUBMC] and Seoul National University Hospital), ischemic stroke patients with active cancer presenting within 72 h of symptom onset from January 2010 to December 2016 were included. Active cancer was defined as a new diagnosis, recurrence, or progression of cancer, or treatment for cancer within 6 months before enrollment, as suggested in previous studies^6,7,11,12,22^. Similar to the protocol at our centers for other stroke patients, ischemic stroke patients with active cancer were hospitalized and underwent broad evaluation, including brain MRI, echocardiography, electrocardiogram, and laboratory examinations to find the etiology and predict the prognosis^6^. Based on the results of these investigations, we selected patients with cryptogenic strokes who were considered to have more cancer-specific mechanisms^1,5,9,13^, excluding conventional mechanisms, such as large artery atherosclerosis, cardioembolism, small vessel occlusion, and other determined etiologies according to the Trial of Org 10172 in the Acute Stroke Treatment classification^25^. Additionally, we excluded patients with the following conditions: (1) age under 18 years, (2) a history of hematologic or primary brain cancer, which has stroke mechanisms different from those observed in solid cancers, (3) absence of brain MRI data, or (4) discharge within 72 h of admission^6^. Altogether, 179 cryptogenic stroke patients with active cancer were included in the analysis.

This two-center retrospective cross-sectional study was approved by the Institutional Review Board (IRB) of SMG-SNUBMC (IRB number: 20-2021-35), which waived the requirement for written informed consent owing to the retrospective nature of the study. We used only de-identified patient information. All experiments were performed in accordance with the tenets of the Declaration of Helsinki and the relevant guidelines and regulations. All data and materials related to this article are included in the main text and supplemental materials.

### Clinical assessments

We evaluated baseline demographic and clinical factors, including age, sex, hypertension, diabetes, dyslipidemia, current smoking status, initial stroke severity, and thrombolytic therapy^6^. The initial stroke severity was rated using the NIHSS score on a daily basis by well-trained neurologists who were not involved in the current study. Characteristics of cancer including types of cancer, systemic metastasis, and adenocarcinoma, were also evaluated^6^. Laboratory examinations, including hemoglobin A1c level, fasting glucose level, total cholesterol level, white blood cell count, hs-CRP level, and D-dimer level were obtained within the first 24 h of admission^6^.

As outcome variables, we assessed END and the 3-month functional status. END was defined as a ≥ 2-point increase in the total NIHSS score or a ≥ 1-point increase in the motor NIHSS score within the first 72 h of admission^6,12^. The 3-month functional status was rated using the mRS score. Based on this score, we divided the participants into two groups: the favorable outcome group (mRS score: 0–2) and the unfavorable outcome group (mRS score: 3–6)^6^.

### Radiological assessments

All participants underwent brain MRI and magnetic resonance angiography (MRA) within 24 h of admission using a 1.5-T MR scanner (Achieva 1.5 T; Philips, Eindohoven, the Netherlands). Details of the MRI scan acquisition were as follows: basic slice thickness = 5.0 mm; diffusion weighted imaging (DWI) [repetition time (TR)/echo time (TE) = 3,000/44 ms]; T1-weighted images (TR/TE = 500/11 ms); T2-weighted images (TR/TE = 3,000/100 ms); fluid-attenuated inversion recovery (FLAIR) images (TR/TE = 11,000/120 ms); T2 gradient echo images (TR/TE = 57/20 ms); and three-dimensional time of flight MRA (TR/TE = 24/3.5 ms, slice thickness = 1.2 mm). The patterns of initial DWI lesions were classified as single or multiple territory lesions^7^. We also evaluated cSVD, including the WMH, SBI, and CMBs subtypes^15^. For WMH, lesions on FLAIR images were measured in the periventricular and subcortical areas, respectively, using the Fazekas scale^26^. As described in a previous study, we combined the Fazekas scales of the two areas and classified the patients into mild (0–2) and severe (3–6) WMH groups based on the scores^26^. SBIs were defined as asymptomatic and well-defined lesions ≥ 3 mm in size, with signal characteristics same as those of the cerebrospinal fluid on T1- or T2-weighted images^15^. CMBs were defined as focal round lesions less than 10 mm in size with low signal on T2 gradient echo images^15^. The burdens of SBI and CMBs were classified as absent, single, or multiple based on the number of lesions^27^. All radiological assessments were rated by two well-trained neurologists (K.-W.N. and Y.-S.L.), and disagreements were resolved through a discussion with a third rater (H.-M.K.).

### Statistical analysis

All statistical analyses were performed using SPSS version 20.0 (IBM Corp., Armonk, NY, USA). Univariate analyses for the evaluation of possible predictors of END were performed using Student’s *t*-test or Mann–Whitney *U*-test for continuous variables and chi-squared or Fisher’s exact test for categorical variables. Based on the results of the univariate analyses, variables with *P* < 0.05 were included in the multivariable logistic regression analysis along with age and sex as confounders (model 1). To further strengthen the association between cSVD and END, we performed an additional multivariable analysis by introducing the cSVD burden as a multi-categorical variable (model 2). Continuous variables with skewed data were transformed into a log scale. All analysis processes were conducted in the same way for unfavorable outcomes, another outcome variable.

Additionally, to determine the mechanism through which cSVD (particularly SBI) affects the functional outcomes, baseline characteristics according to the SBI burden were compared. For this analysis, we used the chi-squared test, Kruskal–Wallis test, and the Jonckheere-Terpstra test. Lastly, we used a relatively sensitive definition of END in this study. To prove its clinical usefulness, we compared the discharge outcomes between the END group and the no-END group, using the discharge NIHSS score, discharge and 3-month mRS scores. All variables with *P* < 0.05 were considered to be statistically significant.

## Supplementary Information

Supplementary Information.

## References

[1] Bang OY (2020). Cancer-related stroke: An emerging subtype of ischemic stroke with unique pathomechanisms. J. Stroke.

[2] Navi BB (2018). New diagnosis of cancer and the risk of subsequent cerebrovascular events. Neurology.

[3] Navi, B. B. *et al.* Cancer and embolic stroke of undetermined source. *Stroke*, STROKEAHA. 120.032002. 10.1161/STROKEAHA.120.032002 PMC7902455 33504187

[4] Navi BB (2017). Risk of arterial thromboembolism in patients with cancer. J. Am. Coll. Cardiol..

[5] Kim SG (2010). Ischemic stroke in cancer patients with and without conventional mechanisms: a multicenter study in Korea. Stroke.

[6] Nam KW (2017). D-dimer as a predictor of early neurologic deterioration in cryptogenic stroke with active cancer. Eur. J. Neurol..

[7] Nam K-W (2017). Predictors of 30-day mortality and the risk of recurrent systemic thromboembolism in cancer patients suffering acute ischemic stroke. PLoS ONE.

[8] Kneihsl M (2016). Poor short-term outcome in patients with ischaemic stroke and active cancer. J. Neurol..

[9] Navi BB (2014). Cryptogenic subtype predicts reduced survival among cancer patients with ischemic stroke. Stroke.

[10] Navi BB, Iadecola C (2018). Ischemic stroke in cancer patients: a review of an underappreciated pathology. Ann. Neurol..

[11] Lee MJ (2017). Hypercoagulability and mortality of patients with stroke and active cancer: the OASIS-CANCER study. J. Stroke.

[12] Nam K-W (2018). Temporal changes in the neutrophil to lymphocyte ratio and the neurological progression in cryptogenic stroke with active cancer. PLoS ONE.

[13] Schwarzbach CJ (2012). Stroke and cancer: the importance of cancer-associated hypercoagulation as a possible stroke etiology. Stroke.

[14] Shin Y-W (2016). Predictors of survival for patients with cancer after cryptogenic stroke. J. Neurooncol..

[15] Wardlaw JM (2013). Neuroimaging standards for research into small vessel disease and its contribution to ageing and neurodegeneration. Lancet Neurol..

[16] Nam K-W (2019). Serum homocysteine level is related to cerebral small vessel disease in a healthy population. Neurology.

[17] Lee E-J, Kang D-W, Warach S (2016). Silent new brain lesions: innocent bystander or guilty party?. J. Stroke.

[18] Appleton JP (2020). Imaging markers of small vessel disease and brain frailty, and outcomes in acute stroke. Neurology.

[19] Baik M (2017). Differential impact of white matter hyperintensities on long-term outcomes in ischemic stroke patients with large artery atherosclerosis. PLoS ONE.

[20] Rost NS (2010). White matter hyperintensity volume is increased in small vessel stroke subtypes. Neurology.

[21] Charidimou A (2016). Cerebral microbleeds and white matter hyperintensities in cardioembolic stroke patients due to atrial fibrillation: single-centre longitudinal study. J. Neurol. Sci..

[22] Ha J (2019). Prevalence and impact of venous and arterial thromboembolism in patients with embolic stroke of undetermined source with or without active cancer. J. Am. Heart Assoc..

[23] Kim BJ, Lee S-H (2015). Prognostic impact of cerebral small vessel disease on stroke outcome. J. Stroke.

[24] Siegler JE, Martin-Schild S (2011). Early Neurological Deterioration (END) after stroke: the END depends on the definition. Int. J. Stroke.

[25] Adams Jr, H. P. *et al.* Classification of subtype of acute ischemic stroke. Definitions for use in a multicenter clinical trial. TOAST. Trial of Org 10172 in Acute Stroke Treatment. *stroke* **24**, 35–41 (1993). 10.1161/01.str.24.1.35 7678184

[26] Nam K-W, Kwon H-M, Lim J-S, Lee Y-S (2017). Leukoaraiosis is associated with pneumonia after acute ischemic stroke. BMC Neurol..

[27] Nam KW, Kwon HM, Kim HL, Lee YS (2019). Left ventricular ejection fraction is associated with small vessel disease in ischaemic stroke patients. Eur. J. Neurol..

