# Supplementary material for: Effects of cerebral small vessel disease on the outcomes in cryptogenic stroke with active cancer
Source: Sci Rep. 2021 Sep 1;11:17510. doi: 10.1038/s41598-021-97154-1 (PMC8410810; doi:10.1038/s41598-021-97154-1)
Supplement: Supplementary file 1 — Supplementary Information. [file 41598_2021_97154_MOESM1_ESM.pdf]

Supplemental information

Supplementary Table 1. Baseline characteristics of the study population (n = 179)

**Demographic & clinical factors**	
Age, y [SD]	67 ± 10
Sex, male, n (%)	108 (60.3)
Hypertension, n (%)	77 (43.0)
Diabetes, n (%)	55 (30.7)
Dyslipidemia, n (%)	76 (42.5)
Current smoking, n (%)	61 (34.1)
Cancer type, n (%)	
Lung	52 (29.1)
Gastric/esophageal	17 (9.5)
Colorectal	10 (5.6)
Hepatobiliary	55 (30.7)
Genitourinary	36 (20.1)
Breast	4 (2.2)
Others	5 (2.8)
Systemic metastasis, n (%)	104 (58.1)
Adenocarcinoma, n (%)	93 (59.2)
Initial NIHSS score [SD]	7 ± 6
Thrombolytic therapy, n (%)	13 (7.3)
**Laboratory factors**	
HbA1c, % [SD]	6.1 ± 0.9
Fasting blood sugar, mg/dL [SD]	107 ± 35
Total cholesterol, mg/dL [SD]	170 ± 52
White blood cell, x 10^3/µL [SD]	8.18 ± 3.47
High-sensitivity CRP, mg/dL [SD]	4.16 ± 5.85
D-dimer, µg/mL [SD]	7.94 ± 12.96
**Radiological factors**	
MRI lesion pattern, n (%)	
Single territory	85 (47.5)
Multiple territory	94 (52.5)
Periventricular WMH (Fazekas scale), [SD]	1 ± 1
Subcortical WMH (Fazekas scale), [SD]	0 ± 1

Silent brain infarct, n (%)	65 (36.3)
Cerebral microbleeds, n (%)	41 (22.9)
**Clinical outcomes**	
Early neurological deterioration, n (%)	31 (17.3)
3-months Modified Rankin Scale [SD]	3 ± 2

NIHSS = National Institutes of Health Stroke Scale, CRP = c-reactive protein, MRI = magnetic resonance imaging, WMH = white matter hyperintensity

Supplementary Table 2. Comparisons of characteristics according to the burden of silent brain infarcts

	Silent brain infarct				
	None	Single	Multiple	*P*-value	*P for trend*
Age	68 [59-73]	70 [60-77]	70 [62-77]	0.125	0.042
Sex	64 (56.1)	13 (50.0)	31 (79.5)	0.019	0.024
Systemic metastasis	66 (57.9)	14 (53.8)	24 (61.5)	0.825	0.773
Adenocarcinoma	58 (55.2)	15 (65.2)	20 (69.0)	0.337	0.149
Initial NIHSS score	3 [2-7]	7 [4-13]	7 [3-14]	0.002	0.001
White blood cell	7.42 [5.75-10.36]	7.33 [5.64-10.29]	8.02 [5.92-9.44]	0.899	0.756
High-sensitivity CRP	1.39 [0.25-5.31]	1.31 [0.58-7.56]	0.87 [0.24-5.27]	0.594	0.858
D-dimer	1.99 [0.70-5.43]	3.01 [0.80-11.84]	5.33 [1.48-18.02]	0.040	0.014
Multiple territory lesion	56 (49.1)	13 (50.0)	25 (64.1)	0.260	0.129

NIHSS = National Institutes of Health Stroke Scale, CRP = c-reactive protein
